# Characteristics, management and outcome of a large necrotising otitis externa case series: need for standardised case definition

**DOI:** 10.1017/S002221512100462X

**Published:** 2022-07

**Authors:** S H Hodgson, V J Sinclair, J Arwyn-Jones, K Oh, K Nucken, M Perenyei, V Sivapathasingam, P Martinez-Devesa, S T Pendlebury, J D Ramsden, P C Matthews, P Pretorius, M I Andersson

**Affiliations:** 1 Department of Infection, Oxford University Hospitals NHS Foundation Trust, Oxford, UK; 2 Department of ENT, Oxford University Hospitals NHS Foundation Trust, Oxford, UK; 3 Department of General (Internal) Medicine and Geratology, Oxford University Hospitals NHS Foundation Trust, Oxford, UK; 4 Neuroradiology, Oxford University Hospitals NHS Foundation Trust, Oxford, UK; 5 Wolfson Centre for Prevention of Stroke and Dementia, Nuffield Department of Clinical Neurosciences, Oxford University, Oxford, UK; 6 Nuffield Division of Clinical Laboratory Science, Radcliffe Department of Medicine, University of Oxford, John Radcliffe Hospital, Oxford, UK; 7 Jenner Institute, Oxford University, Oxford, UK; 8 Nuffield Department of Medicine, University of Oxford, Oxford, UK; 9 Department of ENT, University College London Hospitals NHS Foundation Trust, London, UK; 10 Department of Otolaryngology and Head and Neck Surgery, Nepean Public Hospital, Kingswood, Australia

**Keywords:** Necrotising, Malignant, Otitis, Externa, Pseudomonas, Antimicrobial

## Abstract

**Background:**

Necrotising otitis externa is a severe ear infection for which there are no established diagnostic or treatment guidelines.

**Method:**

This study described clinical characteristics, management and outcomes for patients managed as necrotising otitis externa cases at a UK tertiary referral centre.

**Results:**

A total of 58 (63 per cent) patients were classified as definite necrotising otitis externa cases, 31 (34 per cent) as probable cases and 3 (3 per cent) as possible cases. Median duration of intravenous and oral antimicrobial therapy was 6.0 weeks (0.49–44.9 weeks). Six per cent of patients relapsed a median of 16.4 weeks (interquartile range, 23–121) after stopping antimicrobials. Twenty-eight per cent of cases had complex disease. These patients were older (*p =* 0.042), had a longer duration of symptoms prior to imaging (*p* < 0.0001) and higher C-reactive protein at diagnosis (*p =* 0.005). Despite longer courses of intravenous antimicrobials (23 *vs* 14 days*; p =* 0.032), complex cases were more likely to relapse (*p =* 0.016).

**Conclusion:**

A standardised case-definition of necrotising otitis externa is needed to optimise diagnosis, management and research.

## Introduction

Necrotising otitis externa, also termed malignant otitis externa, is an invasive infection arising in the external auditory canal. It is typically seen in elderly, diabetic or immunocompromised patients.^1,2^ Individuals present with severe otitis externa and associated bone infection. The symptoms of necrotising otitis externa, including severe ear pain and hearing loss, can have a profound effect on quality of life. These patients are at risk of serious complications including cranial nerve palsies and skull-base osteomyelitis. The disease is associated with a high mortality.^1^ One case series reported overall survival of 38 per cent at 5 years with disease-specific mortality of 14 per cent.^3^ An unexplained increase in necrotising otitis externa incidence has been observed both in the UK and elsewhere over the last decade.^4–6^

The management of necrotising otitis externa is also of concern from an antimicrobial stewardship perspective because patients typically receive prolonged courses of empirical, broad-spectrum antimicrobials with limited evidence to support the choice, duration or route of therapy.^1,7^

No established national or international diagnostic or treatment guidelines exist for necrotising otitis externa.^8^ Most published data are limited and of poor quality, consisting of small, retrospective case series and anecdotal experience, where each study applies a different case definition which is often not explicit.^7^ Not surprisingly, the optimal strategy for diagnosis and management of necrotising otitis externa remains uncertain, and there is considerable variability in how this condition is managed.^9^

Here we present a dataset of one of the largest, most detailed unselected retrospective case series of patients with a clinical diagnosis of necrotising otitis externa at a UK teaching hospital over a period of five years.

## Materials and methods

The study was conducted at the Oxford University Hospitals NHS Foundation Trust, a large teaching hospital in the South East of the UK with 1100 in-patient beds and over a million out-patient attendances each year.

Consecutive patients with a clinical diagnosis of necrotising otitis externa documented in their medical records and managed at Oxford University Hospitals NHS Foundation Trust were added retrospectively to a database over a five-year period (August 2013 to July 2018) by two ENT surgeons. Cases were identified by regular review of hospital in-patient lists, specialist clinics and discharge summaries. Typically, the diagnosis was made in cases of refractory otitis externa with granulation tissue in the ear canal requiring admission to hospital. Variables including demographics, co-morbidities, investigations, clinical management and outcome were retrospectively collected and analysed, using both hard copy records and electronic data. One-year mortality data were collected from electronic hospital records.

The study was reviewed by the ‘Joint Research Office Study Classification Group’ at Oxford University Hospitals NHS Foundation Trust and was determined to be a service evaluation assessing the quality of management of necrotising otitis externa. Data were collected and stored in accordance with relevant governance standards.

The current standard of care for necrotising otitis externa in our centre includes a recommendation for sampling for microbiology culture (Supplementary Methods in the supplementary material, available on *The Journal of Laryngology & Otology* website), one to two weeks' intravenous (IV) anti-pseudomonal antibiotics (ceftazidime or piperacillin-tazobactam) followed by a further four weeks of oral ciprofloxacin, in the absence of data to suggest otherwise.

Radiological images were interpreted as consistent with necrotising otitis externa or not at the time of imaging by the duty clinical neuroradiologist as part of standard care.

Markers of frailty (history of falls, Abbreviated Mental Test score less than 9, diagnosis of dementia, Braden score less than 19, Malnutrition Universal Screening Tool score more than 0, delirium or agitation) were obtained via the electronic patient record from assessments completed on admission according to standard Oxford University Hospitals NHS Foundation Trust protocols. Nutritional status and pressure sore risk were taken from routine nursing risk assessments. Cognitive status including abbreviated mental test score (Abbreviated Mental Test score, low score defined as less than 9 of 10), dementia and delirium diagnosis were taken from the Oxford University Hospitals NHS Foundation Trust cognitive screen completed by admitting medical staff.^10^

Risk factors for necrotising otitis externa, as identified in the literature,^1,7,11^ were recorded in the following categories: diagnosis of diabetes, age of 80 years or more, chronic alcohol excess, severe malnutrition, use of immunosuppressive medication, human immunodeficiency virus infection, or recent chemotherapy.^1^

Cases were classified according to the criteria in Table 1. These criteria were derived from review of the relevant literature^1,7,11^ and multidisciplinary discussion at our centre.

**Table 1.** Case definitions

Case type	Definition
Definite case	Defined clinical syndrome AND radiological changes consistent with necrotising otitis externa OR defined clinical syndrome with cranial nerve palsy in the absence of radiological changes consistent with necrotising otitis externa
Probable case	Defined clinical syndrome without cranial nerve palsy AND risk factors in the absence of radiological changes consistent with necrotising otitis externa
Possible case	Defined clinical syndrome without cranial nerve palsy with no risk factors & the absence of radiological changes consistent with necrotising otitis externa

Defined clinical syndrome = otalgia and the presence of granulation tissue in the external auditory canal on the affected side.

Patients with cranial nerve palsies, skull base osteomyelitis, involvement of the temporomandibular joint (TMJ), cerebral venous sinus thrombosis or spread of disease contralaterally were defined as having complex disease, irrespective of bony changes on imaging. Relapse was defined as a diagnosis of necrotising otitis externa more than 14 days after stopping a prescribed antimicrobial course, with progressive radiological changes, occurring within 1 year of stopping antimicrobial therapy.

Statistical analysis to investigate associations was performed using the Mann–Whitney test for continuous variables and Fisher's exact test for categorical variables. Statistical analysis was performed using GraphPad Prism statistical software (GraphPad Software, Version 9.0.2, San Diego, USA). A two-tailed alpha value of less than 0.05 was considered significant.

## Results

**Fig. 1.**
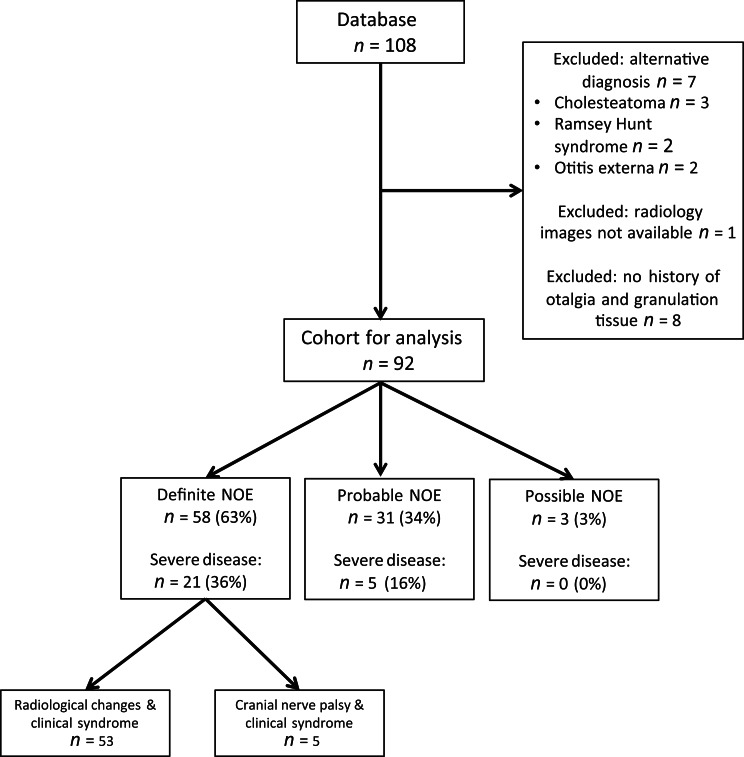
Consort diagram of patients meeting eligibility criteria for inclusion in the study. NOE = necrotising otitis externa

A total of 108 patients were identified over a 5-year period. Seven patients were excluded because a diagnosis other than necrotising otitis externa was subsequently confirmed (cholesteatoma *n =* 3, Ramsey Hunt syndrome *n =* 2, otitis externa *n =* 2) (Figure 1). Radiological images were not available for one patient whose imaging was performed at another centre, and this patient was excluded from further analysis. Eight patients with neither a history of otalgia nor granulation tissue in the external auditory canal were also excluded. The remaining 92 patients had imaging performed at presentation, 91 had computed tomography (CT) imaging and one patient only had a magnetic resonance imaging (MRI) scan. Nine patients (10 per cent) had both CT and MRI scans performed at presentation. No other forms of imaging were performed. Of the 92 cases, 58 (63 per cent) were definite cases, 31 (34 per cent) were probable cases and 3 (3 per cent) were possible cases of necrotising otitis externa.

### Demographics and clinical presentation of patients

Of the 92 patients (Figure 1), 53 patients were male (58 per cent) with a median age of 85 years (range, 20–104 years) (Table 2). While the majority were elderly, 8 patients were under 60 years old, including 3 patients aged 20, 28 and 57 years old for whom no risk factors for necrotising otitis externa were identified. Although 91 per cent (84 of 92) of patients had at least one risk factor for necrotising otitis externa, only 60 per cent (55 of 92) of patients were diabetic.

**Table 2.** Clinical characteristics, clinical presentation, surgical and antimicrobial management, and outcome for all cases

Parameter	Case definition	All cases	Definite	Probable	Possible	*P*-value (definite *vs* probable cases)
Patients (*n*)		92	58	31	3	
Complex disease (%)		28	36	16	0	0.054
Demographics & risk factors	– Median age (years)	85	84	85	74	0.167
	– Age range (years)	20–104	20–96	58–104	72–78	
	– Male (%)	58	62	48	66	0.263
	– Ear syringing* (% (*n*))	60 (23/39)	47 (9/19)	72 (13/18)	50 (1/2)	0.184
	– Risk factors for NOE (%)	91	91	100	0	0.159
	– Diabetic (%)	60	64	58	0	0.085
Signs and symptoms at presentation	– Otalgia (%)	100	100	100	100	1.0
	– Night pain (% (*n*))	87 (48/55)	91 (31/34)	84 (16/19)	50 (1/2)	0.655
	– Otorrhoea (% (*n*))	89 (71/80)	90 (45/50)	87 (27/31)	100	0.726
	– Hearing Loss (% (*n*))	97 (77/79)	96 (48/50)	100 (26/26)	100	0.544
	– Feverishness (% (*n*))	9 (6/70)	11 (5/47)	5 (1/20)	0	0.662
	– Any CN palsy (%)	22	34	0	0	<0.0001^†,^**
	– VIIth CN palsy (% (*n*))	20	31 (18/58)	0	0	<0.0001^†,^**
	– Granulation tissue (%)	100	100	100	100	1.0
	– Polyp (% (*n*))	79 (63/80)	76 (37/49)	86 (24/28)	66	0.386
Investigations at presentation	– Duration of symptoms prior to imaging (median/range; days) (*n* = 76)	30 (1–224)	37 (1–700)	25 (7–140)	29 (28–120)	0.081
	– WCC × 10^9^/l (median/range; WCC × 10^9^/l) (*n* = 91)^‡^	8.2 (0.5–36.0)	8.6 (0.5–36.0)	7.0 (4.6–18.4)	7.2 (5.3–7.2)	0.009**
	– CRP (median/range; mg/l) (*n* = 88)^‡^	15 (0–152)	19 (0–152)	12 (1–124)	9 (2–10)	0.483
	– ESR (median/range; mm/hour) (*n* = 45)^‡^	34 (2–116)	37 (2–116)	20 (5–87)	18 (9–26)	0.631
Surgery	– Debridement of granulation tissue (% (*n*))	78 (71/91)	75 (43/57)	81 (25/31)	100	0.791
	– Surgery in operating theatre (% (*n*))	8 (7/88)	9 (5/55)	7 (2/30)	0	>0.999
Antimicrobial management	– Percentage receiving IV AMs (% (*n*))	98 (89/91)	98 (56/57)	98 (30/31)	100	>0.999
	– Duration IV AMs (median/range; days) (*n* = 89)	14 (1–68)	16 (1–68)	11 (2–42)	14 (2–28)	0.013**
	– Duration of total AMs (median/range; weeks) (*n* = 88)	6.0 (0.9–44.9)	6.1 (0.9–44.9)	6.0 (1–16.7)	4.0 (2.3–5.0)	0.087
	– Percentage treated with topical AMs (% (*n*))	95 (80/84)	92 (46/50)	100	100	0.292
	– Duration topical AMs (median/range; days) (*n* = 70)	26 (3–81)	29 (3–74)	23 (4–81)	14 (14)	0.312
	– Length of in-patient stay (median/range; days) (*n* = 91)	14 (1–87)	15 (1–87)	10 (2–44)	6 (2–14)	0.035**
Outcome	– One-year mortality (% (*n*))	19 (17/90)	25 (14/57)	10 (3/30)	0	0.155
	– Relapse of disease (% (*n*))	6 (5/84)	7 (4/54)	4 (1/27)	0	0.660

*Ear syringing in community in four months preceding diagnosis; ^†^the presence of a cranial nerve palsy was part of the case definition for definite disease; ^‡^laboratory values were taken within 48 hours of diagnosis; **statistically significant. Where data for a variable were not available for all patients, the denominator is shown. NOE = necrotising otitis externa; CN = cranial nerve; WCC = white cell count; CRP = C-reactive protein; ESR = erythrocyte sedimentation rate, IV = intravenous; AMs = antimicrobials

**Fig. 2.**
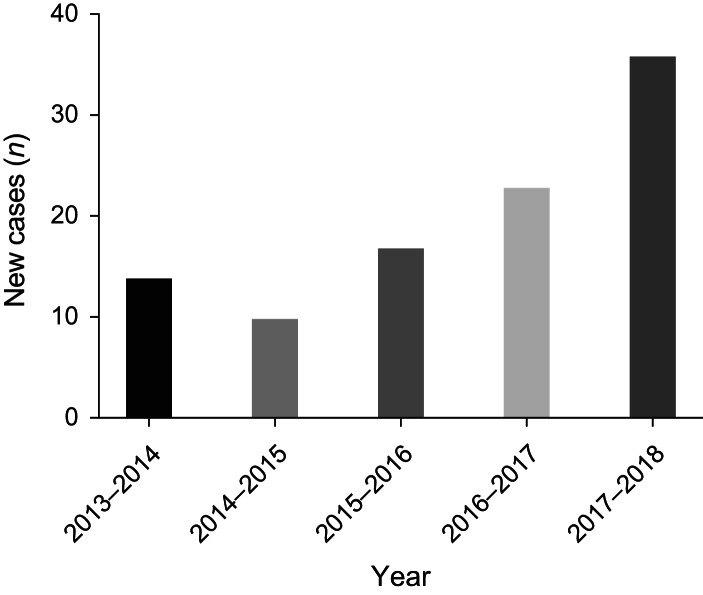
Numbers of new cases presenting each year (August to July).

We observed an increase in number of cases diagnosed each year (Figure 2), with more cases presenting in the winter months (see Figure 1 in the supplementary material, available on *The Journal of Laryngology & Otology* website). Among patients for whom data were available, 60 per cent (23 of 39) had ear syringing in the 4 months preceding diagnosis (median, 34 days; range, 14–116 days).

Markers of frailty were prevalent. Thirty-nine per cent (29 of 74) of patients had at least one frailty marker (see Figure 2 in the supplementary material, available on *The Journal of Laryngology & Otology* website). There was no significant difference in the numbers of patients with at least one frailty marker between definite and probable cases (*p =* 0.469, Fisher's exact test) or patients with or without complex disease (*p =* 0.585, Fisher's exact test) (supplementary Figure 2).

Presenting features in cases included night pain, otorrhoea and hearing loss, affecting 87 per cent (48 of 55), 89 per cent (71 of 80) and 97 per cent (77 of 79) of patients, respectively (Table 2). A small proportion of patients (9 per cent, 6 of 70) reported feverishness on presentation. There was no significant difference in presentation between definite and probable cases (Table 2).

### Clinical characteristics of patients with complex disease

Complex disease was present in 26 patients (28 per cent), among whom 81 per cent (21 of 26) were classified as definitely having necrotising otitis externa. Of these cases, 5 (19 per cent) were classified as definite necrotising otitis externa because of the presence of a lower cranial nerve palsy and defined clinical syndrome. Nineteen per cent of patients with complex disease (5 of 26) were classified as probable cases of necrotising otitis externa. These patients had TMJ involvement (4 of 5 patients) or skull base osteomyelitis (1 of 5 patients) but no external auditory canal bony erosion visible on CT scan.

Patients with complex disease were significantly older than those without complex disease (88 *vs* 83 years; *p =* 0.042, Fisher's exact test), but there was no significant difference in frailty markers between the two groups (supplementary Figure 2).

Patients with complex disease had a longer duration of symptoms prior to imaging compared with those patients without (65 *vs* 28 days; *p* < 0.0001, Mann–Whitney test). However, there was no difference in symptoms and signs between patients with complex disease and those without (see Table 1 in the supplementary material, available on *The Journal of Laryngology & Otology* website).

### Blood markers for diagnosis and assessment

White cell count differentiated definite from probable cases (median, 8.6 × 10^9^/l *vs* 7.0 × 10^9^/l, respectively, *p* = 0.009, Mann–Whitney test) (Table 2, and Figure 3 in the supplementary material, available on *The Journal of Laryngology & Otology* website). There was a significant difference in C-reactive protein (CRP) at diagnosis between patients with complex disease and those without (median, 23.2 mg/l *vs* 9.8 mg/l respectively, *p* = 0.005, Mann–Whitney test, supplementary Table 1 and supplementary Figure 3). These markers were not elevated in all cases, suggesting normal inflammatory markers cannot exclude a diagnosis of necrotising otitis externa.

### Organisms identified

Organisms were identified in 70 per cent (62 of 89) of sampled patients**.** *Pseudomonas aeruginosa* was the most commonly identified organism, cultured in 58 per cent (52 of 89) of sampled patients. Quinolone resistance in *P aeruginosa* isolates was identified in 3 of 45 isolates (7 per cent) on *in vitro* testing.

### Differences in treatment duration

Ninety-eight per cent of patients (89 of 91) received IV anti-pseudomonal antibiotics for a median of 14 days (range, 1–68 days) (Table 2). Duration of IV antimicrobial therapy was longer for definite cases compared with probable cases (16 days *vs* 11 days; *p* = 0.013, Mann–Whitney test) and in those patients with complex disease compared with those without complex disease (23 days *vs* 14 days; *p =* 0.032, Mann–Whitney test, supplementary Table 1).

Most patients (86 per cent, 78 of 91) had a course of oral antimicrobial therapy after the IV anti-pseudomonal treatment. Oral ciprofloxacin monotherapy was prescribed for the majority of patients (70 per cent, 64 of 91). A proportion of patients (15 per cent, 14 of 91) received dual oral antimicrobial follow on therapy, and the choice of agents was not clearly determined by culture results. Only one patient, who had extensive disease, received treatment with an anti-fungal agent.

The median total duration of antimicrobial therapy (IV and oral) was 6.0 weeks (interquartile range, 5.7–8.3 weeks). Ninety-five per cent of patients (80 of 84) received topical antimicrobial therapy with a median duration of therapy of 26 days (range, 3–81 days). Ciprofloxacin was the topical antimicrobial used most commonly (52 per cent, 44 of 84). Forty-four per cent (37 of 84) of patients receiving topical antimicrobials had these co-administered with a topical steroid.

### In-patient stay

Patients had extended in-patient stays (median, 14 days; range, 1–87) despite availability of an out-patient parenteral antibiotic therapy service. Definite cases had longer in-patient admissions than probable cases (15 days *vs* 10 days; *p =* 0.035, Mann–Whitney test). Similarly, patients with complex disease spent longer in hospital than those without complex disease (20 days *vs* 10 days; *p =* 0.0001, Mann–Whitney test). The infection team provided input into the care of 87 per cent of patients (78 of 90) and were more likely to be involved in the care of definite compared with probable cases (*p =* 0.047, Fisher's exact test).

### Differences in outcome

Relapse of disease was seen in 6 per cent of patients (5 of 84), who re-presented at a median of 16.4 weeks (interquartile range, 23–121 weeks) after stopping antimicrobial therapy. There was no significant difference in relapse rates between definite and probable cases of necrotising otitis externa (*p =* 0.712, Fisher's exact test) or patients with diabetes or without diabetes (*p =* 0.388, Fisher's exact test). However, complex cases were more likely to present with relapse (*p =* 0.016, Fisher's exact test). The 1-year all-cause mortality was 19 per cent for the cohort. Disease attributable mortality data were not available.

## Discussion

This series reports one of the largest and most detailed retrospective cohorts of patients who were clinically defined and managed as having necrotising otitis externa. We describe a heterogenous group of patients, reflecting those commonly presenting to clinical services, for whom the lack of diagnostic and treatment guidelines makes management challenging. Consistent with previous data,^4,5^ our series shows an increase in the number of cases diagnosed with necrotising otitis externa over time, adding impetus to the need for high-quality research in this neglected area.

### Definition of cases

There are no widely accepted, published criteria for the diagnosis of necrotising otitis externa.^7^ Published case series often lack clearly defined inclusion criteria^6,12,13^ or use criteria which could exclude true cases, such as requiring the isolation of pseudomonas on microbiological sampling.^14,15^ The lack of a ‘gold standard’ case definition for necrotising otitis externa limits comparison of patient characteristics and natural history across observational studies and precludes comparison of outcomes of interventional studies.

### Role of radiology

There is consistent agreement in the literature that imaging is an important part of the diagnostic approach for suspected cases of necrotising otitis externa.^1,7,16,17^ However, there is debate regarding the best first-line imaging modality to use and the utility of routine MRI or isotope-labelled imaging.^16–18^ Although technetium scans have been advocated by some^3,19^ and demonstrate bone involvement earlier than CT,^20^ given that most institutions only have access to CT scanners, it is rational to base diagnostic algorithms on CT images in the first instance. We believe external auditory canal bone erosion on CT, in the presence of a fitting clinical syndrome, is consistent with a diagnosis of osteomyelitis of the temporal bone and therefore necrotising otitis externa. However, given that external auditory canal bone erosion may not be visible on CT in the first weeks of symptoms^20^ and, as our series demonstrates, external auditory canal bone changes may be absent on CT even in complex cases of necrotising otitis externa, CT alone may be insufficiently sensitive to identify all cases of necrotising otitis externa. Further work is needed to better understand the role of different imaging modalities in the diagnosis and follow up of these cases.

### Risk factors

Our data support the notion that necrotising otitis externa typically occurs in the elderly, diabetic or immunocompromised individuals.^1^ The median age of patients was 85 years, and the majority were male (58 per cent). Although 91 per cent of patients had at least one risk factor for necrotising otitis externa, it is of interest that only 60 per cent of patients were diabetic. On a population level, diabetes is an important risk factor for necrotising otitis externa. A recent large case control study in Taiwan showed the adjusted odds ratio of patients with diabetes for necrotising otitis externa was 10.07 (95 per cent confidence interval, 8.15 to 12.44).^21^

Consistent with previous data,^22^ we found that 60 per cent of all cases (for whom data were available), had ear syringing in the 4 months prior to diagnosis. The UK national guidance on the community management of hearing loss because of ear wax does not provide any caution regarding the removal of ear wax in those most at risk of necrotising otitis externa.^23^

### Complex cases

In our cohort, 28 per cent of patients had complex disease. Our large series allowed us to demonstrate that patients with complex disease were older, had a longer duration of symptoms prior to diagnosis and were more likely to experience relapse. It is unclear whether these patients presented to any clinical service later or whether primary care clinicians were more likely to try empirical treatments in these patients, delaying referral to specialist services for assessment and investigation.

In contrast to a report in the early necrotising otitis externa literature,^24^ our data showed that elevated CRP, not erythrocyte sedimentation rate was significantly associated with complex disease. However, there was a large overlap in CRP values between those with complex and non-complex disease suggesting this assay cannot reliably distinguish one group from the other. Neither CRP nor erythrocyte sedimentation rate were useful in identifying those with definite necrotising otitis externa.

Importantly, 19 per cent of patients with complex disease were classified as probable cases of necrotising otitis externa only because of absence of supportive radiological changes or cranial nerve palsy. This highlights the need for imaging to be interpreted in a clinical context and suggests that bone erosion of the external auditory canal is not always demonstrable on imaging or may resolve in some cases while other pathology progresses. These important cases highlight a limitation of our case definition. Further studies are needed to examine these cohorts in greater detail.

### Microbiology

A total of 58 per cent of cases in our cohort cultured *P aeruginosa* on ear swab, and 7 per cent of *P aeruginosa* isolates in our series were resistant to quinolones, restricting this group of patients to long-term parenteral therapy. Increasing pseudomonas resistance to quinolones is being reported in patients with necrotising otitis externa,^25,26^ and this could potentially become a greater problem for these elderly, co-morbid patients.

### Antimicrobial therapy

A total of 98 per cent of patients were treated with IV antimicrobial therapy effective against pseudomonas species. In contrast to other centres, dual anti-pseudomonal antimicrobial therapy was not used.^27^ A considerable proportion of patients were treated with combination oral antimicrobial therapy after IV therapy (30 per cent), likely reflecting clinician concern that *P aeruginosa* may not be solely responsible for the disease.^1,6,12^

The median duration of antimicrobial therapy was six weeks, which is the shortest duration of antimicrobial therapy for the treatment of necrotising otitis externa reported in a survey of UK ENT surgeons.^9^ Topical antimicrobial therapies were used in 95 per cent of patients, in addition to IV and oral antimicrobial therapy, with courses up to 81 days in duration. Although in line with current UK practice, there is no evidence to demonstrate the efficacy of topical antimicrobial use in necrotising otitis externa.^9^ The attendant potential risk of facilitating the evolution of ciprofloxacin resistance may be an additional reason to avoid using topical antibiotics.^25,26^

The use of extended courses of broad-spectrum antibiotics is not without risk. The Food and Drug administration (FDA) and the European Medicine Agency have both issued alerts about the risks of long-term fluroquinolone use in the elderly.^28,29^

### Outcomes

In our cohort, 6 per cent of patients relapsed, which was lower than the mean rate of 9.6 per cent reported in a recent review.^7^ Of note, the likelihood of relapse was not associated with age, diabetes or duration of antimicrobial therapy. However, patients with complex disease were more likely to relapse, despite receiving longer courses of parenteral antimicrobial therapy. Research suggests that increasing duration of antimicrobials in chronic bone and joint infections prolongs the interval to relapse rather than reducing the likelihood of relapse itself.^30^ The same may be true for necrotising otitis externa.

### Limitations

The strength of this study is the large number of cases of an uncommon condition seen in a single centre, but the results should be interpreted in the context of its limitations. The dataset was retrospectively collected, and the sample size was not large enough for detailed statistical analyses. The major limitation of our study was the potential misclassification of cases, a result of the absence of a well-established, widely accepted definition for necrotising otitis externa. We have initiated a national Delphi-style process of specialists in ENT, infection and radiology in the UK to agree definitions for this condition to facilitate future research.

- Necrotising otitis externa is a severe ear infection with no widely accepted guidelines for diagnosis or treatment
- This study is one of the largest unselected, retrospective cohorts of patients with necrotising otitis externa to date
- Six per cent of patients relapsed at a median of 16.4 weeks after stopping antimicrobials
- Complex disease was common in older patients, who had a longer duration of symptoms and higher C-reactive protein at diagnosis
- Despite longer courses of intravenous antimicrobials, complex cases were more likely to relapse
- A widely accepted, standardised case definition for necrotising otitis externa is needed

## Conclusion

This work presents one of the largest cohorts of clinical cases of necrotising otitis externa described to date. Our data show that necrotising otitis externa commonly, though not exclusively, occurs in elderly, vulnerable, immunocompromised patients. Despite prolonged courses of anti-pseudomonal antimicrobial therapy, with the associated significant side effects, relapse occurs relatively frequently.

There is an imperative to work on improving outcomes for patients with this infection. This will require active collaboration between centres to pool expertise, establish a consensus case definition and design high-quality prospective studies to understand how to manage this infection, optimising patient outcomes while simultaneously reducing the risks of antimicrobial overuse.

## Data Availability

The full dataset is available from the Figshare repository at: https://doi.org/10.6084/m9.figshare.14219714.v1

## Supplementary material

For supplementary material accompanying this paper visit https://doi.org/10.1017/S002221512100462X. click here to view supplementary material
