# Supplementary material for: Characteristics, management and outcome of a large necrotising otitis externa case series: need for standardised case definition
Source: J Laryngol Otol. 2022 Jan 19;136(7):604–10. doi: 10.1017/S002221512100462X (PMC9257435; doi:10.1017/S002221512100462X)
Supplement: Supplementary file 1 [file S002221512100462Xsup001.doc]

<JLO 13472; supplementary material>

**Supplementary Methods**. Processing of microbiological samples

Samples were processed according to local laboratory standard operating procedures in accordance with the UK Standards for Microbiological Investigations SMI (standards for microbiological investigations) B1: ‘Investigation of ear infections and associated samples’.^31^

All superficial swab samples were processed using the BD Kiestra^TM^ automated system. Each superficial sample was inoculated onto a haemolysed blood agar plate incubated in carbon dioxide, a blood agar plate incubated anaerobically, a MacConkey agar plate incubated aerobically and a Columbia naladixic acid agar plate incubated aerobically. Plates that showed no growth were then re-incubated for a further 24 hours and examined at 48 hours.

Tissue samples were processed in a Class II cabinet in the microbiology laboratory. A total of 5 mL saline with Ballotini balls were added to the specimen and vortexed for 15 seconds. Each sample was then inoculated: into a chopped meat broth; onto a blood agar plate incubated in O2, a blood agar plate incubated anaerobically and BD CHROMagar Orientation Medium (ORI, Becton Dickinson, USA) incubated aerobically. Agar plates were read after 24 hours of incubation. Plates that showed no growth were then re-incubated for a further 24 hours (except the ORI chrome agar) and examined at 48 hours. The cooked meat was routinely sub-cultured after 24 hours incubation onto a blood agar incubated anaerobically and a haemolysed blood agar plate incubated in carbon dioxide and read as other plates.

Samples were reported as ‘no growth’ if no growth was seen on the plates. Samples were reported as ‘no significant growth’ if only typical skin contaminants were cultured (including enterococci, coagulase-negative staphylococci and diphtheroid corynebacterium). Isolates from cooked meat only were reported as ‘from enrichment only’.

Samples culturing pseudomonas were only sub-cultured for antimicrobial sensitivity testing if the sample request specified the sample was from a diabetic patient, or related to a diagnosis of otitis externa or necrotising otitis externa.

Any bacterial identification was performed using Matrix assisted laser desorption/ionisation – time of flight mass spectrometry. Antimicrobial sensitivities were performed using the BD Phoenix^TM^ automated susceptibility system or by manual antimicrobial sensitivity testing method as appropriate depending on the organism.

<Refhead>

References

<Reftext>

1 Rubin Grandis J, Branstetter BF, Yu VL. The changing face of malignant (necrotising) external otitis: clinical, radiological, and anatomic correlations. *Lancet Infect Dis* 2004;**4**:34–9

2 Sylvester MJ, Sanghvi S, Patel VM, Eloy JA, Ying YM. Malignant otitis externa hospitalizations: analysis of patient characteristics. *Laryngoscope* 2017;**127**:2328–36

3 Stern Shavit S, Soudry E, Hamzany Y, Nageris B. Malignant external otitis: factors predicting patient outcomes. *Am J Otolaryngol* 2016;**37**:425–30

4 Chawdhary G, Liow N, Democratis J, Whiteside O. Necrotising (malignant) otitis externa in the UK: a growing problem. Review of five cases and analysis of national Hospital Episode Statistics trends. *J Laryngol Otol* 2015;**129**:600–3

5 Hopkins ME, Bennett A, Henderson N, MacSween KF, Baring D, Sutherland R. A retrospective review and multi-specialty, evidence-based guideline for the management of necrotising otitis externa. *J Laryngol Otol* 2020;**134**:487–92

6 Kumar SPR, A. Somu, L. Ismail, N.M. Malignant otitis externa: an emerging scourge. *J Clin Gerontol Geriatr* 2013;**4**:128–31

7 Mahdyoun P, Pulcini C, Gahide I, Raffaelli C, Savoldelli C, Castillo L *et al*. Necrotizing otitis externa: a systematic review. *Otol Neurotol* 2013;**34**:620–9

8 Hasibi M, Ashtiani MK, Motassadi Zarandi M, Yazdani N, Borghei P, Kuhi A *et al*. A treatment protocol for management of bacterial and fungal malignant external otitis: a large cohort in Tehran, Iran. *Ann Otol Rhinol Laryngol* 2017;**126**:561–7

9 Chawdhary G, Pankhania M, Douglas S, Bottrill I. Current management of necrotising otitis externa in the UK: survey of 221 UK otolaryngologists. *Acta Otolaryngol* 2017;**137**:818–22

10 Pendlebury ST, Lovett NG, Smith SC, Dutta N, Bendon C, Lloyd-Lavery A *et al*. Observational, longitudinal study of delirium in consecutive unselected acute medical admissions: age-specific rates and associated factors, mortality and re-admission. *BMJ Open* 2015;**5**:e007808

11 Rubin J, Yu VL. Malignant external otitis: insights into pathogenesis, clinical manifestations, diagnosis, and therapy. *Am J Med* 1988;**85**:391–8

12 Bhat V, Aziz A, Bhandary SK, Aroor R, Kamath PS, Saldanha M. Malignant otitis externa - a retrospective study of 15 patients treated in a tertiary healthcare center. *J Int Adv Otol* 2015;**11**:72–6

13 Peled C, Kraus M, Kaplan D. Diagnosis and treatment of necrotising otitis externa and diabetic foot osteomyelitis - similarities and differences. *J Laryngol Otol* 2018;**132**:775–9

14 Lambor DV, Das CP, Goel HC, Tiwari M, Lambor SD, Fegade MV. Necrotising otitis externa: clinical profile and management protocol. *J Laryngol Otol* 2013;**127**:1071–7

15 Levenson MJ, Parisier SC, Dolitsky J, Bindra G. Ciprofloxacin: drug of choice in the treatment of malignant external otitis (MEO). *Laryngoscope* 1991;**101**:821–4

16 Morales RE, Eisenman DJ, Raghavan P. Imaging necrotizing otitis externa. *Semin Roentgenol* 2019;**54**:215–26

17 van Kroonenburgh A, van der Meer WL, Bothof RJP, van Tilburg M, van Tongeren J, Postma AA. Advanced imaging techniques in skull base osteomyelitis due to malignant otitis externa. *Curr Radiol Rep* 2018;**6**:3

18 Mehrotra P, Elbadawey MR, Zammit-Maempel I. Spectrum of radiological appearances of necrotising external otitis: a pictorial review. *J Laryngol Otol* 2011;**125**:1109–15

19 Cohen D, Friedman P. The diagnostic criteria of malignant external otitis. *J Laryngol Otol* 1987;**101**:216–21

20 Lau K, Scotta G, Wu K, Kabuli MAK, Watson G. A review of thirty-nine patients diagnosed with necrotising otitis externa over three years: is CT imaging for diagnosis sufficient? *Clin Otolaryngol* 2020;**45**:414–8

21 Yang TH, Xirasagar S, Cheng YF, Wu CS, Kao YW, Shia BC *et al*. Malignant otitis externa is associated with diabetes: a population-based case-control study. *Ann Otol Rhinol Laryngol* 2020;**129**:585–90

22 Rubin J, Yu VL, Kamerer DB, Wagener M. Aural irrigation with water: a potential pathogenic mechanism for inducing malignant external otitis? *Ann Otol Rhinol Laryngol* 1990;**99**:117–9

23 NICE. Hearing loss in adults: assessment and management. 2018. https://www.nice.org.uk/guidance/ng98 [20 March 2022]

24 Rubin J, Stoehr G, Yu VL, Muder RR, Matador A, Kamerer DB. Efficacy of oral ciprofloxacin plus rifampin for treatment of malignant external otitis. *Arch Otolaryngol Head Neck Surg* 1989;**115**:1063–9

25 Bernstein JM, Holland NJ, Porter GC, Maw AR. Resistance of pseudomonas to ciprofloxacin: implications for the treatment of malignant otitis externa. *J Laryngol Otol* 2007;**121**:118–23

26 Rehman A, Patrick WM, Lamont IL. Mechanisms of ciprofloxacin resistance in pseudomonas aeruginosa: new approaches to an old problem. *J Med Microbiol* 2019;**68**:1–10

27 Pulcini C, Mahdyoun P, Cua E, Gahide I, Castillo L, Guevara N. Antibiotic therapy in necrotising external otitis: case series of 32 patients and review of the literature. *Eur J Clin Microbiol Infect Dis* 2012;**31**:3287–94

28 Administration USFD. FDA warns about increased risk of ruptures or tears in the aorta blood vessel with fluoroquinolone antibiotics in certain patients. 2018. https://www.fda.gov/drugs/drug-safety-and-availability/fda-warns-about-increased-risk-ruptures-or-tears-aorta-blood-vessel-fluoroquinolone-antibiotics [20 March 2022]

29 EMA. Disabling and potentially permanent side effects lead to suspension or restrictions of quinolone and fluoroquinolone antibiotics. 2018. https://www.ema.europa.eu/en/news/disabling-potentially-permanent-side-effects-lead-suspension-restrictions-quinolone-fluoroquinolone [20 March 2022]

30 Byren I, Bejon P, Atkins BL, Angus B, Masters S, McLardy-Smith P *et al*. One hundred and twelve infected arthroplasties treated with 'DAIR' (debridement, antibiotics and implant retention): antibiotic duration and outcome. *J Antimicrob Chemother* 2009;**63**:1264–71

31 PHE. UK Standards for Microbiology Investigations. 2014. In: https://www.gov.uk/government/collections/standards-for-microbiology-investigations-smi [20 March 2020]**[Q2]**

**Fig. 1.** Incidence of necrotising otitis externa by month of diagnosis

**
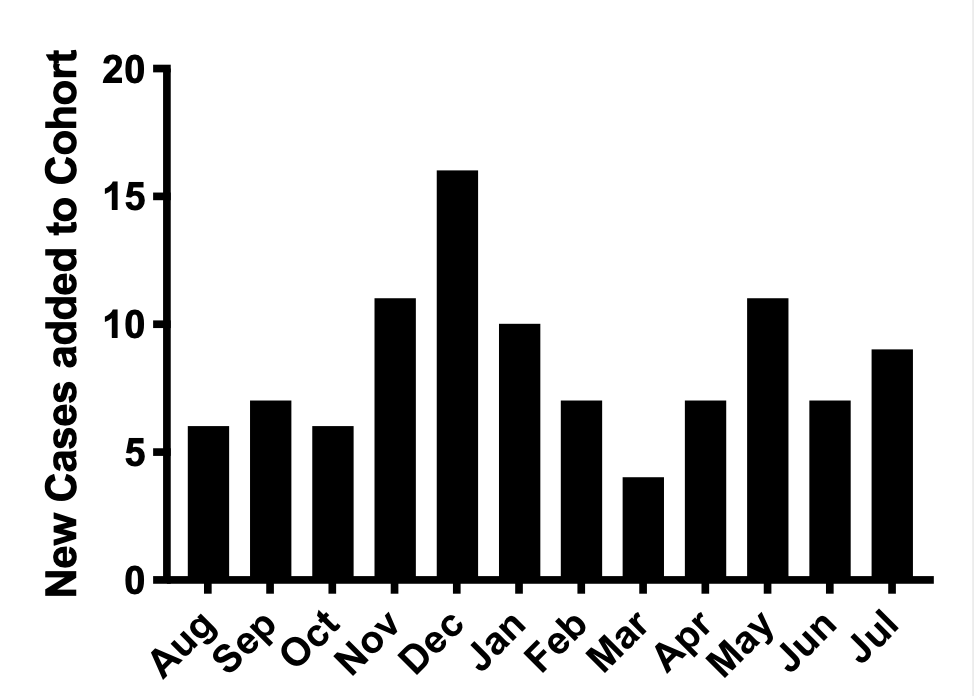
**

**–** Please add x-axis label ‘Month’.

– Please make y-axis label sentence case and add ‘(*n*)’ at the end of the y axis label.

**Fig. 2.** Frailty markers of patients

(a) Frailty markers for patients according to case definition. (b) Frailty makers for patients according to complexity of disease. AMTS = Abbreviated Mental Test score; MUST = Malnutrition Universal Screening Tool

**Fig. 3.** Laboratory measures of infection

Measures of white cell count (WCC), C reactive protein (CRP) and erythrocyte sedimentation rate (ESR) according to case definition (a–c) and complexity of disease (d–f). All samples were taken within 48 hours of diagnosis. Median values are indicated with a bar. ***p* < 0.01.

**Table 1. Clinical characteristics, clinical presentation, surgical and antimicrobial management and outcome for complex and non-complex cases**

| Parameter | Case definition | Complex disease | Non-complex disease | *P*-value |
| --- | --- | --- | --- | --- |
| Patients (*n*) |  | 26 | 66 |  |
| Demographics & risk factors | – Median age (years) | 88 | 83 | 0.042 |
|  | – Age range (years) | 71–96 | 20–104 |  |
|  | – Male (%) | 73 | 52 | 0.066 |
|  | – Ear syringing* (% (*n*)) | 57 (4/7) | 59 (19/32) | >0.999 |
|  | – Risk factors for NOE (%) | 96 | 89 | 0.433 |
|  | – Diabetic (%) | 50 | 64 | 0.247 |
| Signs & symptoms at presentation | – Left side affected (% (*n*)) | 54 (14/26) | 53 (35/66) | >0.999 |
|  | – Ear Pain (%) | 100 | 100 |  |
|  | – Night pain (% (*n*)) (*n* = 55) | 85 (11/13) | 88 (37/42) | 0.664 |
|  | – Aural discharge (*n* = 80) | 78 (18/23) | 93 (53/57) | 0.112 |
|  | – Hearing loss (% (*n*)) (*n* = 79) | 100 (23/23) | 96 (54/56) | >0.999 |
|  | – Feverishness (% (*n*)) (*n* = 70) | 11 (2/19) | 8 (4/51) | 0.660 |
|  | – Any CN palsy (%) (*n* = 92) | 80 | 0 | <0.001^†^,** |
|  | – VIIth CN palsy (%) (*n* = 92) | 69 | 0 | <0.001^†^,** |
|  | – Granulation tissue (%) (*n* = 92) | 100 | 100 |  |
|  | – Polyp (% (*n*)) (*n* = 80) | 78 (18/23) | 79 (45/57) | >0.999 |
| Investigations at presentation | – Duration of symptoms prior to imaging (median/range; days) (*n* = 76) | 65 (3–224) | 28 (1–700) | <0.0001** |
|  | – Previous imaging for NOE (%) (*n* = 92) | 23 | 14 | 0.348 |
|  | – WCC (median/range; WCC × 10^9^/l) (*n* = 91)^‡^ | 8.6 (0.5–36.0) | 8.2 (4.4–18.4) | 0.251 |
|  | – CRP (median /range; mg/l) (*n* = 88)^‡^ | 23 (0–124) | 10 (0–152) | 0.005** |
|  | – ESR (median/range; mm/hour) (*n* = 45)^‡^ | 39 (2–116) | 26 (2–101) | 0.282 |
| Surgery | – Debridement of granulation tissue (% (*n*)) (*n* = 91) | 80 (20/25) | 77 (51/66) | 0.300 |
|  | – Surgery in operating theatre (% (*n*)) (*n* = 88) | 17 (4/23) | 5 (3/65) | 0.073 |
| Antimicrobial management | – Percentage receiving IV AMs (% (*n*)) | 100 | 97 (63/65) | >0.999 |
|  | – Duration IV AMs (median/range; days) (*n* = 89) | 23 (4–68) | 14 (1–58) | 0.032** |
|  | – Duration of total AMs (median/range; weeks) (*n* = 88) | 7.7 (0.9–24) | 6.0 (1.0–44.9) | 0.058 |
|  | Percentage treated with topical AMs (% (*n*)) | 95 (21/22) | 95 (55/58) | >0.999 |
|  | – Duration topical AMs (median/range; days) (*n* = 70) | 18 (3–73) | 26 (4–81) | 0.174 |
|  | – Length of in-patient stay (median/range; days) (*n* = 91) | 20 (5–86) | 10 (1–87) | 0.0001** |
| Outcome | – One-year mortality (% (*n*)) (*n* = 90) | 31 (8/26) | 14 (9/64) | 0.080 |
|  | – Relapse of disease (% (*n*)) (*n* = 84) | 18 (4/22) | 2 (1/62) | 0.016** |

*Ear syringing in community in four months preceding diagnosis; ^†^the presence of a cranial nerve palsy was part of the case definition for definite disease; ^‡^laboratory values were taken within 48 hours of diagnosis; **statically significant. Where data for a variable were not available for all patients the denominator is shown. NOE = necrotising otitis externa; CN = cranial nerve palsy; WCC = white cell count; CRP = C-reactive protein; ESR = erythrocyte sedimentation rate, IV = intravenous; AMs = antimicrobials
